# Childhood Cognitive Ability Moderates Later-Life Manifestation of Type 2 Diabetes Genetic Risk

**DOI:** 10.1037/hea0000184

**Published:** 2015-01-19

**Authors:** René Mõttus, Michelle Luciano, John M. Sarr, Mark I. McCarthy, Ian J. Deary

**Affiliations:** 1 Centre for Cognitive Ageing and Cognitive Epidemiology and Department of Psychology, University of Edinburgh, and Department of Psychology, University of Tartu; 2 Centre for Cognitive Ageing and Cognitive Epidemiology, University of Edinburgh; 3 Centre for Cognitive Ageing and Cognitive Epidemiology, University of Edinburgh and Geriatric Medicine Unit, Western General Hospital, Edinburgh, United Kingdom; 4 Oxford Centre for Diabetes, Endocrinology and Metabolism, Wellcome Trust Centre of Human Genetics, Churchill Hospital, University of Oxford; 5 Centre for Cognitive Ageing and Cognitive Epidemiology and Department of Psychology, University of Edinburgh

**Keywords:** cognitive ability, intelligence, diabetes, HbA11c, genetic risk

## Abstract

***Objective:*** The study investigated whether childhood cognitive ability moderates Type 2 diabetes polygenic risk manifestation in older age. ***Method:*** In 940 relatively healthy people (mean age 69.55 ± 0.85), we tested whether self-reported diabetes and hemoglobin HbA1c (HbA1c) levels were predicted by diabetes polygenic risk, cognitive ability measured about 60 years earlier, and their interaction. Polygenic risk scores aggregated the small effects of up to nearly 121,000 single-nucleotide polymorphisms (SNPs). Participants’ cognitive ability was measured at age 11. ***Results:*** Both polygenic risk and low childhood cognitive ability significantly predicted diabetes diagnosis. Polygenic risk interacted with cognitive ability (*p* = .02), predicting HbA1c levels more strongly in people with below-median cognitive ability (effect *r* = .21) than in people with above-median cognitive ability (effect *r* = .10). The interaction term was not significant for self-reported diabetes (*p* = .34), although the genetic risk-diabetes association showed a tendency of being stronger among those with below-median cognitive ability. ***Conclusions:*** Higher premorbid cognitive ability may provide some environmental protection against the manifestation of Type 2 diabetes genetic risk. This information may improve early identification of diabetes risk and inform intervention development.

The recent increase in the prevalence of diabetes is projected to continue world-wide (Wild, Roglic, Green, Sicree, & King, 2004), underscoring the importance of better understanding its pathogenesis and early risk factors. This study investigates whether the stable behavioral trait of cognitive ability moderates the manifestation of polygenetic risk for Type 2 diabetes in older age.

Type 2 diabetes (T2D) and its diagnostic marker hemoglobin HbA1c (HbA1c) demonstrate substantial heritability (Almgren et al., 2011; Simonis-Bik et al., 2008). Genome-wide association studies (GWAS) have discovered several common genetic variants that account for about 10% of the total genetic effects on T2D (Morris et al., 2012). For the rest, a large number of common variants, or variants in linkage disequilibrium with those on genotyping chips, may be implicated, with each having effects too small to be detected by the current GWAS projects (Stahl et al., 2012). Given this possibility, however, existing GWAS data can be used to quantify polygenic risk for such complex diseases by aggregating the (often statistically nonsignificant by conventional standards) small effects of thousands of variants (cf. Davies et al., 2011; Purcell et al., 2009; Yang et al., 2010).

Yet, probably not everyone genetically predisposed to T2D develops the condition. Pointing to gene-environment interactions (Franks, Pearson, & Florez, 2013), T2D genetic risk may be moderated by dietary habits (Nettleton et al., 2010; Qi, Cornelis, Zhang, van Dam, & Hu, 2009) and physical activity may interact with the genetic risk of obesity, a T2D precursor (Andreasen et al., 2008; Kilpeläinen et al., 2011). Here, we take a more general view by considering the moderating role of general cognitive ability, a factor possibly underlying diabetes-prone lifestyle choices (Deary, Weiss, & Batty, 2010). Lower childhood cognitive ability predicts diabetes-related outcomes such as metabolic syndrome (Batty et al., 2008), obesity (Batty, Deary, Schoon, & Gale, 2007a), and inflammation (Luciano, Marioni, Gow, Starr, & Deary, 2009), as well as later diabetes itself (Olsson, Hulting, & Montgomery, 2008). These links may be mediated by lifestyle choices, as lower cognitive ability is associated with smoking (Taylor et al., 2003), poorer diet and less physical activity (Batty, Deary, Schoon, & Gale, 2007b), and less persistent adhering to medical treatment (Deary, Gale, et al., 2009). This is the rationale for hypothesizing that cognitive ability may also moderate the links between TD2 genetic risk and its actual manifestation. Besides theoretical implications of such a possible moderation, cognitive ability can be reliably measured many decades before the typical T2D onset age and may therefore contribute to early identification of people at risk.

## Method

### Sample

Almost all of the 1,091 community-dwelling members of the Lothian Birth Cohort 1936 (LBC1936) sat for an intelligence test in 1947 (at about age 11) and were followed up between 2004 and 2007. Details on the background, recruitment, and testing procedures are available elsewhere (Deary et al., 2007). The present study uses data from those 940 LBC1936 members (481 males; mean age 69.55, *SD* = 0.85) for whom complete data on childhood cognitive ability, self-reported diabetes, HbA1c, and T2D poly genetic risk were available. Participants provided written informed consent. Ethics permissions were obtained from the Multi-Centre Research Ethics Committee for Scotland.

### Measures

Age-11 cognitive ability was measured using the Moray House Test No. 12 that included word classification, arithmetic and spatial items, and proverbs. Demonstrating concurrent validity, it correlated about 0.80 with the Terman-Merrill Revision of the Binet Test (Deary, Whalley, & Starr, 2009). During follow-up testing at the clinic, participants were asked about their diabetes status and their HbA1c levels were measured. Participants were genome-wide genotyped and their T2D polygenic risk was quantified by combining these data with previously published meta-analytic results (Morris et al., 2012) on the association between T2D and nearly 121,000 single-nucleotide polymorphisms (SNPs). For each participant, the meta-analytic effect size of each SNP’s “risk” allele was multiplied by the number of copies of the risk allele carried by the participant. The sums of these individual risks across all SNPs formed participants’ T2D polygenic risk scores. For robustness analysis, seven additional risk scores were calculated by applying increasingly stringent inclusion criteria for SNPs. See Supplemental Material for further details.

## Results

Of the 940 participants, 83 (8.8%) reported diabetes (11 of them reported insulin therapy, suggesting possible Type 1 diabetes), whereas 117 (12.4%) had HbA1c levels equal to or above the cut-off of diabetes diagnosis (The International Expert Committee, 2009). See Supplemental Material for further details and analyses. Higher T2D polygenic risk significantly predicted self-reported diabetes (odds ratio per standard deviation increase in predictor [*OR*] = 1.81, 95% CI [1.40, 2.34], *p* < .001). People with higher childhood cognitive ability were less likely to report diabetes (*OR* = 0.72, 95% CI [0.59, 0.89], *p* < .001). Higher T2D polygenic risk also significantly predicted higher HbA1c levels (standardized linear regression weights [β] = .16, 95% CI [.09, .22], *p* < .001), as did lower childhood cognitive ability (β = −.13, 95% CI [−.19, −.06], *p* < .001).

**Figure 1 fig1:**
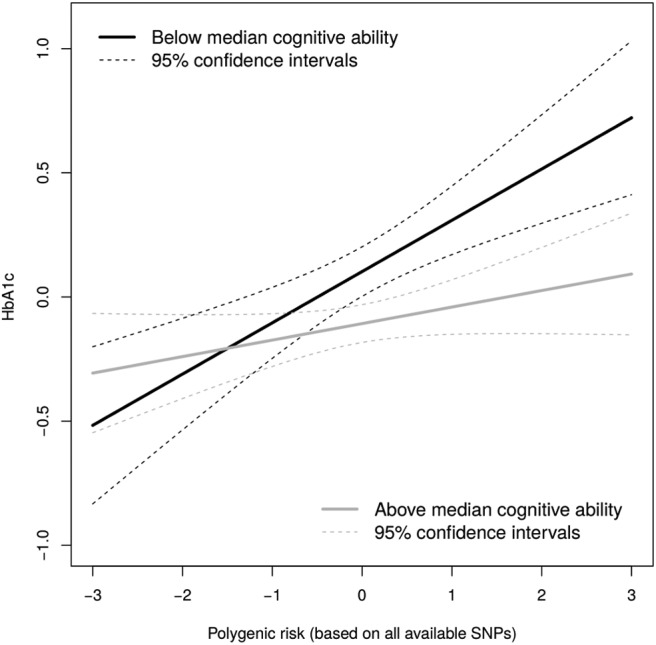
The interaction between polygenic risk and childhood cognitive ability in the prediction of HbA1c.

The interaction term between childhood cognitive ability and polygenic risk was statistically nonsignificant (*p* = .34) when predicting self-reported diabetes status, but significant (*p* = .02) for the prediction of HbA1c. When people were split at median into those with lower and higher childhood cognitive ability (see Figure 1), the risk scores predicted HbA1c levels significantly more strongly in the lower cognitive ability group (β = .21, 95% CI [.11, .31], *p* < .001) than in the higher ability group (β = .10, 95% CI [.01, .19], *p* = .04; see also Supplemental Material for an alternative splitting). The nonsignificant interaction in the prediction of self-reported diabetes was in the same direction: the association between genetic risk and diagnosis was stronger in those below median cognitive ability (*OR* = 1.93, 95% CI [1.38, 2.74], *p* < .001) than among those with higher ability (*OR* = 1.64, 95% CI [1.10, 2.49], *p* < .05). These findings tended to replicate across the alternative risk scores based on fewer SNPs (see Supplemental Material for details). There was little evidence for the association being driven by participants with potential Type 1 diabetes or by the use of hypoglycemic drugs (see Supplementary Material for details). Finally, we found negligible evidence for the role of body mass index (BMI) in polygenic risk-diabetes association or in the polygenic risk-cognitive ability interaction (see Supplemental Material for details).[Fig-anchor fig1]

## Discussion

The study presented novel evidence for a genetic risk by stable behavioral trait interaction: polygenic risk for T2D was more likely to manifest in elevated diabetes marker HbA1c at age 70 among people with lower childhood cognitive ability than in those with higher ability test performance nearly 60 years earlier.

There are at least two possible reasons for why the interaction was not significant, although in the same direction, when self-reported diabetes diagnosis was used as an outcome. First, with the sample containing just 83 (8.8%) people with reported diabetes diagnosis, statistical power to detect a significant interaction for the binary outcome was limited, whereas the power was higher for continuously defined HbA1c. Second, diabetes might have been underdiagnosed in the present sample because the proportion of people with HbA1c above the cut-off was higher (12.4%) than the proportion of self-reported diabetes. As a result, self-reported diabetes could have been a relatively less accurate measure of actual diabetes status than HbA1c.

By showing that there may be identifiable factors that facilitate or lessen the links between genetic disease risk and actual disease phenotype, these findings extend the existing few previous reports of gene-environment interactions in T2D (Kilpeläinen et al., 2011; Nettleton et al., 2010; Qi et al., 2009). Low cognitive ability is a known antecedent of many unhealthy lifestyle choices (Deary et al., 2010). It is therefore plausible that the mechanisms by which lower cognitive ability can alter genetic risks include health-related behaviors such as dietary habits and exercise, and smoking (Taylor et al., 2003) or health-management (Deary, Gale, et al., 2009) may also play a role. Also, higher cognitive ability is known to contribute to educational attainment and, as a result, higher socioeconomic success (Deary & Johnson, 2010), which may also, by means of better physical and social environments, lessen the effects of genetic risk. However, the possible mediating role of BMI in the polygenic risk-diabetes association (and it being moderated by cognitive ability) was found to be small, suggesting that the mechanisms by which genetic risk for TD2 manifests itself, and the moderating role of cognitive ability in these mechanisms, may be more complex than was proposed above. Thus, although BMI was measured only concurrently with the diabetes whereas life-course BMI may have provided more relevant information for studying diabetes development, the mechanism of how cognitive ability might facilitate diabetes remain elusive.

The present results have possible practical implications in at least two important ways. First, to behave in ways that help to buffer the genetic risks, people do not necessarily have to *have* high cognitive ability as such. It may very well suffice if people with lower cognitive ability *mimic* what their higher-ability peers do differently with respect to diabetes-related behaviors. This is akin to what has previously been termed phenocopying, whereby an individual’s environmentally produced phenotypic characteristics are the same as those normally produced by genetic factors (Deary et al., 2010). The targets of phenocopying may include dietary habits and exercise but also smoking and health care habits. Of course, such health lifestyle choices could and should be encouraged in everyone, regardless of their cognitive ability levels, which brings us to the second implication.

Second, it is possible that not all people react to health interventions in the same way. In particular, it has been suggested that people with lower cognitive ability are less likely to benefit from different types of public or targeted health interventions than their cognitively more able peers (Gottfredson, 2004). For example, there is evidence that public campaigns on various issues are more likely to reach people with higher education—and therefore, higher cognitive abilities—than people with lower education (Weenig & Midden, 1997). As a result, to the extent that there are specific and potentially modifiable lifestyle aspects that may facilitate diabetes genetic risk manifestation, they are likely to operate on people’s broader cognitive background. It may be useful to factor this background into any planned interventions. In other words, although it undeniably is a good idea to promote healthy behaviors in all people to buffer against any possible genetic risk turning into actual disease phenotype, such interventions may be informed by understanding the antecedents of the behaviors to be targeted and thereby the barriers that may interfere with changing them. For example, if and when there are indications that the people or groups of people to be targeted with an intervention may have somewhat lower verbal or reasoning abilities (e.g., low educational level), then the interventions may need to be delivered in ways that are appropriately tailored. Our results empirically corroborate that the cognitive background is indeed relevant in genetic risk manifestation and not knowing or ignoring this possibility is not likely to benefit anyone.

For most people, the relative level of cognitive ability is highly stable across most of the life-course (Deary, Whalley, Lemmon, Crawford, & Starr, 2000). This means that cognitive ability level can be used as a predictor of health outcomes at various points across life-course, including several decades before the typical onset age of diseases such as T2D (as was done in this study). Because the same is true for genetic differences between people, individuals’ cognitive ability and genetic makeup can be used in combination to more accurately identify people at higher diabetes risk several decades before the likely onset age of the disease itself. This may open possibilities for early prevention strategies. Of course, often cognition-related information may not be readily available, in which case proxy variables such as school performance or educational attainment may prove useful.

The findings are potentially informative in terms of diabetes pathogenesis and prevention but they may also have implications for the field of genetic risk prediction more generally. Specifically, it is possible that including behavioral traits such as cognitive ability may also improve the accuracy of the genetic prediction of other conditions. From a purely psychological point of view, it is likewise interesting that a well-established psychological trait may manifest in people’s lives by moderating how genetic predispositions for health conditions become manifest. It is possible that such gene by trait interactions exist for other health phenotypes and involve other psychological traits as well.

Finally, an important limitation of the study is the sample size, which was relatively modest for a genetic risk prediction study. Therefore, these findings should and are intended to be replicated in larger studies that have relevant information (e.g., the United Kingdom’s National Child Development Study [the 1958 British Birth Cohort] or U.K. Biobank).

## Supplementary Material

10.1037/hea0000184.supp
