# Supplementary material for: Childhood Cognitive Ability Moderates Later-Life Manifestation of Type 2 Diabetes Genetic Risk
Source: Health Psychol. 2015 Jan 19;34(9):915–9. doi: 10.1037/hea0000184 (PMC4562329; doi:10.1037/hea0000184)
Supplement: Supplementary file 1 [file zg1-3111_HEA-2013-1784_SUPPL.zip › zg1-3111_HEA-2013-1784_SUPPL/zg1001153111so1.pdf]

SUPPLEMENTAL MATERIAL

Childhood cognitive ability moderates the later-life manifestation of type 2 diabetes genetic risk

René Mõttus, Michelle Luciano, John M, Starr, Mark M. McCarthy, and Ian J. Deary.

Methods

Materials and Analyses

Cognitive ability. Scores of Moray House Test no. 12, sat in 1947 when participants were about 11 years old, were adjusted for age at time of testing and converted to standard scores with mean of 0 (*SD* = 1). The predictive and concurrent validity of the test has been extensively documented (Deary, Whalley, & Starr, 2009; Deary, Whiteman, Starr, Whalley, & Fox, 2004). The rankings of individuals in the test scores are highly stable up to the tenth decade of life (Deary, Pattie, & Starr, 2013).

Diabetes. In addition to self-reported diabetes, venous blood samples were taken from participants and their HbA1c levels were measured using the Menarini HA8160 analyser. The HbA1c values were converted to standard scores for further analyses.

Medication. During the interview at clinic, participants were asked to list all the medications they were taking at the time. Insulin and oral hypoglycemic agents were coded as control variables.

Body Mass Index (BMI). Participants' height and weight at age 70 were measured at the clinic by a trained nurse. BMI = mass (kg) / height^2 (m).

Polygenic risk scores. Genome-wide genotyping of the Lothian Birth Cohort 1936 (LBC1936) members was conducted by the Genetics Core Laboratory at the Wellcome Trust Clinical Research Facility, Western General Hospital, Scotland, using the Illumina Human610-Quadv1 Chip. Genotype quality control procedures have been reported elsewhere (Davies et al., 2011). Standard checks for gender discrepancies, individual relatedness, and non-Caucasian descent were performed and necessary exclusions were made (Davies et al., 2011).

For creating the Type 2 diabetes (T2D) polygenic risk scores of the LBC1936 participants, results of an independent, previously published meta-analyses on the association between T2D and single-nucleotide polymorphisms (SNPs) were used (Morris et al., 2012). The meta-analysis was based on multiple cohorts, which together comprised 34,840 T2D cases and 114,981 controls of mostly European descent (Morris et al., 2012). Alleles from all available SNPs were employed (see Table S1 for the number of SNPs) for which associations with diabetes had been calculated in this meta-analysis and for which information existed in the LBC1936 participants. That is, all available genetic information was used regardless of the strength or statistical significance of the associations involving individual SNPs. The assumption of using this method is that a huge number of polymorphisms may contribute to diabetes phenotype, although the existing GWAS studies have not been able to detect these associations (possibly due to being underpowered). This approach has proven to be useful in quantifying the genetic propensity for a number of diseases or traits including schizophrenia (Purcell et al., 2009), height (Yang et al., 2010), and measured cognitive ability (Davies et al., 2011). In computing the risk scores, each SNP's 'risk' allele effect size [logarithm (base 10) of odds ratio] was taken from the above-referred meta-analysis. Then, for each participant

of the present study, this effect size was multiplied by the number of copies (0, 1, or 2) of the risk allele carried by the individual. The sum of these individual risks across all included SNPs formed the polygenetic T2D risk score to be used in further analyses. SNPs that were missing for any individual were imputed based on the observed allele frequency in the cohort. Linkage disequilibrium pruning (based on $r^2 < .25$ within a 200-SNP sliding window) and exclusion of SNPs with minor allele frequency < .02 and with Hardy-Weinberg Equilibrium test p < .001 was done prior to polygenic risk score calculation, reducing the available number of SNPS to 120,991 (because missing SNPs were imputed, all scores were based on this value). The risk scores were calculated using PLINK software (Purcell et al., 2007) and were converted to standard scores; they had normal-like distributions.

For robustness analysis, a series of seven additional risk scores were calculated by applying different thresholds for the *p*-values of the associations of SNPs with T2D found in the above-referred meta-analysis: $p < .05$, $p < .1$, $p < .2$, $p < .3$, $p < .4$, $p < .5$, $p \leq 1$ (see Table S1 for details).

In all analyses involving the polygenic risk scores, the numbers of observed (before imputation) SNPs used in the calculation of the particular risk score for the particular people were used as a co-variate in order to control for any potential systematic relationship between polygenic scores and amount of observed information contributing to the scores (Purcell et al., 2009). Likewise, population stratification estimates (the first four multidimensional scaling components described in Davies et al., 2011) were included as co-variates, along with sex and age at the time of the diabetes/HbA1c measurement. This was done because population stratification, age and sex could have confounded the associations of interest.

Table S1. *Descriptive statistics of the study variables.*

	N	Mean	SD
Sex (0 = male, 1 = female)	940	0.49	0.50
Age	940	69.55	0.85
Self-reported diabetes (0 = no, 1 = yes)	940	0.09	0.28
HbA1c (%)	940	5.94	0.74
Polygenic risk score (p ≤ 1, #SNPs)	940	120991	–
Polygenic risk score (p < .5; #SNPs)	940	62668	–
Polygenic risk score (p < .4; #SNPs)	940	50761	–
Polygenic risk score (p < .3; #SNPs)	940	38782	–
Polygenic risk score (p < .2; #SNPs)	940	26691	–
Polygenic risk score (p < .1; #SNPs)	940	14354	–
Polygenic risk score (p < .05; #SNPs)	940	7224	–
Polygenic risk score (p < .01; #SNPs)	940	1691	–
Body mass index (BMI; kg/m^2)	939	27.83	4.36

NOTE: SD = standard deviation; HbA1c = glycated hemoglobin; # SNPs = the number of SNPs included in the calculation of the respective risk score (because missing SNPs were imputed in the course of calculations, the risk scores of all participants were calculated using the same SNPs). All polygenic risk scores had means of 0 and standard deviations of 1.

Results

Diabetes Status

Eighty three (8.83%) of the 904 participants reported having diabetes. Of them, 11 (13.25%) were on self-reported insulin therapy and 39 (46.99%) on oral hypoglycemic agents, whereas 33 (39.76%) did not report taking any medication for diabetes. The raw hemoglobin HbA1c values (%) ranged from 4.50 to 11.60, with 117 people (12.4%) having levels equal to or above the 6.5% (48 mmol/mol), the cut-off used for diabetes diagnosis (The International Expert Committee, 2009). There were thus more people with biochemical diabetes than with self-reported diabetes diagnosis. A closer look revealed that 70 (84.3%) of those with self-reported diabetes had HbA1c ≥ 6.5%, whereas 47 (5.5%) of those without self-reported diabetes had HbA1c ≥ 6.5%. This could reflect a number of undiagnosed diabetes cases and could mean that measured HbA1c level was a more reliable indicator of diabetes status than self-report. Among those with HbA1 ≥ 6.5%, sex or age did not differentiate significantly between those with and without self-reported diabetes, neither did childhood cognitive ability. For more power and higher discrimination, HbA1c was used as a continuous variable in main analyses.

No Evidence for Pleiotropy

The correlation between childhood cognitive ability and the polygenic risk score was low and non-significant ($r = -.03$, $p = .33$), suggesting no pleiotropy between diabetes and early cognitive ability.

A More Fine-Grain Look at the Interactions

It was tested if the interaction between childhood cognitive ability and polygenic risk was driven by more extreme groups of cognitive ability by dividing the sample into four ability groups (splitting on its quartiles) instead of two. The standardized regression coefficients predicting HbA1c levels from genetic risk scores were as follows (from the lowest cognitive ability group to the highest): $\beta = .26$ ($p < .001$), $\beta = .17$ ($p = .001$), $\beta = .05$ ($p = .51$), and $\beta = .15$ ($p = .03$). The 95% confidence intervals of these coefficients overlapped in all cases. Thus, the moderating effect may not have been linear: the risk tended to be the strongest in the lowest cognitive ability group, whereas the differences were not systematic among the remaining three groups. However, it is noted that analyzing the associations separately in four groups resulted in insufficient statistical power for such expectedly small effect sizes and therefore one should interpret the comparisons based on these four groups very cautiously.

Robustness Analysis

The robustness of these findings was tested by additionally employing seven less 'inclusive' polygenic risk scores: instead of using all available SNPs, these included only those SNPs that had been associated with T2D at $p < .5, .4, .3, .2, .1, .05$, and .01 in the diabetes GWAS consortium data (Morris et al., 2012). The pattern of findings (see Table S2) was broadly consistent across the different polygenic risk scores, although the risk score based on the $p < 0.1$ threshold had non-significant ($p = .13$) interaction term with cognitive ability in predicting HbA1c. The predictions were consistently stronger among people with lower cognitive ability.

Excluding Possible Type 1 Diabetes Cases

It was tested if the interaction could have been driven by cases with possible type 1 diabetes by differentiating between those with and without self-reported insulin medication. The pattern of findings tended to be similar when the 11 participants reporting the use of insulin were excluded, with six of the eight interaction terms being significant at $p < .05$ (for the risk score based on $p < .1$ criterion, the interaction p-value was .14 and for the risk score based on $p < .01$ criterion it was .09). In all cases, the risk score-HbA1c associations were stronger among people with lower cognitive abilities. For example, for the most inclusive risk score, the association with HbA1c was $\beta = .20$ among those with below median cognitive abilities, whereas it was $\beta = .10$ among those with above median cognitive abilities. Therefore, although some reduction in the significance of the interaction term as a result of dropping people with self-reported insulin medication was expected because of the loss of statistical power due to reduced number of people with high HbA1c levels, there was not much evidence for the interaction being driven by the few cases of possible type 1 diabetes.

It is noted, however, that insulin medication is not a non-optimal criterion for classifying people as having type 1 diabetes. Some people with T2D may need insulin, too. Furthermore, the probability of having type 1 diabetes at age 70 is reduced by the fact that this condition is associated with an average life expectancy that is more than 20 years shorter than the population average (Department of Health, 2001).

No Evidence for the Role of Hypoglycemic Medication

Bearing in mind that HbA1c levels may have been influenced by hypoglycemic medication, the analyses were reran controlling for this variable. This additional co-variate did not influence the main findings in any important way. The most inclusive risk score predicted HbA1c levels with $\beta = .11$ ($p < .001$) and the interaction term between the risk score and childhood cognitive ability was significant ($p < .001$; this was consistent across all eight risk scores). As without controlling for hypoglycemic medication, the association was stronger among those with below-median cognitive ability ($\beta = .16$, $p < .001$) than among those with higher cognitive ability ($\beta = .06$, $p = .06$; the pattern was consistent across all risk scores).

Possible Role of Body Weight

Concurrent body mass index (BMI) was considered as a potential mediator in polygenic risk-diabetes association. Including BMI as a co-variate in the models that predicted self-reported diabetes from the polygenic risk scores resulted only in a relatively small (3.5%) reduction of effect size; for HbA1c, the respective reductions were only slightly larger (12.5%). The interaction term between childhood cognitive ability and the polygenic risk score (based on all available SNPs) when predicting HbA1c remained statistically significant ($p = 0.04$). When HbA1c was predicted from the risk scores separately in the low and high cognitive ability groups, the attenuation was quite similarly modest in the low (13.8%) and high cognitive ability (6.1%) groups. These findings were, again, relatively similar for less SNP-inclusive risk scores. Therefore, concurrent BMI did not substantially mediate (or confound) the association between T2D polygenic risk scores and diabetes and it did not account for the interaction between these associations and childhood cognitive ability, although its role in the cognitive ability-diabetes associations appeared slightly larger.

The Interaction from Another Angle

The interaction could also reflect a moderation effect of genetic risk on the prediction of HbA1c from cognitive ability, if low cognitive ability and high genetic risk contribute to diabetes in a

multiplicative manner (i.e., by facilitating each other's effects). Indeed, when people were divided (based on median split using the risk scores based on all available SNPs) into low and high polygenic risk groups, lower childhood cognitive ability predicted HbA1c levels at age 70 more strongly in people at higher genetic risk for type 2 diabetes (β = -.17, $p < .001$) than in people at lower risk for the disease (β = -.08, $p < .05$). This means that low cognitive ability in childhood was more likely to predict high HbA1c when it was coupled with genetic predisposition for diabetes, whereas low genetic risk may have buffered the potentially negative effect of low ability.

Table S2. *Risk scores predicting self-reported diabetes status (binary variable) and HbA1c (continuous variable) along with the statistical significance of their interaction terms with childhood cognitive ability.*

	Self-reported diabetes status		HbA1c	
	OR [95% CI]	Interaction with cognitive ability (*p*-value)	β [95% *CI*]	Interaction with cognitive ability (*p*-value)
Polygenic risk score ($p \leq 1$)	1.81 [1.40; 2.34]	.34	.16 [.09; .22]	.02
Polygenic risk score ($p < .5$)	1.77 [1.38; 2.29]	.22	.16 [.09; .22]	.01
Polygenic risk score ($p < .4$)	1.74 [1.35; 2.25]	.16	.15 [.09; .22]	< .01
Polygenic risk score ($p < .3$)	1.66 [1.30; 2.15]	.34	.15 [.09; .22]	.01
Polygenic risk score ($p < .2$)	1.67 [1.31; 2.14]	.29	.15 [.09; .22]	.02
Polygenic risk score ($p < .1$)	1.55 [1.22; 1.99]	.65	.13 [.07; .20]	.13
Polygenic risk score ($p < .05$)	1.57 [1.24; 2.02]	.12	.14 [.07; .20]	.02
Polygenic risk score ($p < .01$)	1.68 [1.33; 2.15]	.37	.11 [.05; .18]	.04

NOTE: OR = odds ratio; CI = 95% confidence interval; β = standardized regression coefficient. All odds ratios and betas are significant at p < 0.001.

References

Davies, G., Tenesa, A., Payton, A., Yang, J., Harris, S. E., Liewald, D., … Deary, I. J. (2011). Genome-wide association studies establish that human intelligence is highly heritable and polygenic. *Molecular Psychiatry*, *16*, 996–1005. doi:10.1038/mp.2011.85

Deary, I. J., Whalley, L. J., & Starr, J. M. (2009). *A lifetime of intelligence: Follow-up studies of the Scottish mental surveys of 1932 and 1947.* Washington D. C.: American Psychological Association.

Deary, I. J., Whiteman, M. C., Starr, J. M., Whalley, L. J., & Fox, H. C. (2004). The impact of childhood intelligence on later life: Following up the Scottish Mental Surveys of 1932 and 1947. *Journal of Personality and Social Psychology*, *86*, 130–147. doi:10.1037/0022-3514.86.1.130

Department of Health. (2001). *National Service Framework for Diabetes.* Department of Health:

London, UK.
Morris, A. P., Voight, B. F., Teslovich, T. M., Ferreira, T., Segrè, A. V., Steinthorsdottir, V., … DIAbetes Genetics Replication And Meta-analysis (DIAGRAM) Consortium. (2012). Large-scale association analysis provides insights into the genetic architecture and pathophysiology of type 2 diabetes. *Nature Genetics*, *44*, 981–990. doi:10.1038/ng.2383
Purcell, S. M., Neale, B., Todd-Brown, K., Thomas, L., Ferreira, M. A. R., Bender, D., … Sham, P. C. (2007). PLINK: a tool set for whole-genome association and population-based linkage analyses. *American Journal of Human Genetics*, *81*, 559–575. doi:10.1086/519795
Purcell, S. M., Wray, N. R., Stone, J. L., Visscher, P. M., O'Donovan, M. C., Sullivan, P. F., … Sklar, P. (2009). Common polygenic variation contributes to risk of schizophrenia and bipolar disorder. *Nature*, *460*, 748–752. doi:10.1038/nature08185
The International Expert Committee. (2009). International expert committee report on the role of the A1C assay in the diagnosis of diabetes. *Diabetes Care*, *32*, 1327–1334. doi:10.2337/dc09-9033
Yang, J., Benyamin, B., McEvoy, B. P., Gordon, S., Henders, A. K., Nyholt, D. R., … Visscher, P. M. (2010). Common SNPs explain a large proportion of the heritability for human height. *Nature Genetics*, *42*, 565–569. doi:10.1038/ng.608
